# Food and dietary supplement expenditures associated with dietary self-management among women with endometriosis: A cross-sectional study in Hungary

**DOI:** 10.1016/j.pmedr.2026.103652

**Published:** 2026-09-28

**Authors:** Linda Balpataki, Dora B. Balogh, Gernot Hudelist, Christian M. Becker, Andrew W. Horne, Dominika Miklos, Nandor Acs, Attila Bokor

**Affiliations:** a Corvinus University of Budapest, Center for Foreign Language Education and Research, 8 Fővám Square, Budapest 1093, Hungary; b Semmelweis University, Department of Obstetrics and Gynecology, 27 Baross Street, Budapest 1088, Hungary; c Hospital St. John of God, Department of Gynecology- Center for Endometriosis, 1 Johannes-von-Gott-Platz, Vienna 1020, Austria; d University of Oxford, Oxford Endometriosis CaRe Centre, Nuffield Department of Women's and Reproductive Health, John Radcliffe Hospital, Headington, Oxford OX39DU, UK; e University of Edinburgh, Centre for Reproductive Health, Institute for Regeneration and Repair, Edinburgh BioQuarter, 4-5 Little France Drive, Edinburgh EH16 4UU, UK

**Keywords:** Endometriosis, Cost of illness, Diet, Dietary supplements, Self-management

## Abstract

**Objective:**

To compare the financial burden of special diets and dietary supplement use in women with and without endometriosis.

**Methods:**

This cross-sectional, mobile health app–based survey in Hungary (December 2023–August 2024; 90% response rate) evaluated 1645 women aged 14–45 years (524 with endometriosis, 1121 without). Primary outcomes were monthly household food and individual supplement expenditures. Secondary outcomes included diet adherence and supplement use.

**Results:**

Women with endometriosis, particularly those following special diets, reported significantly higher food and supplement spending than reference participants (*p* < 0.01). Despite smaller households (mean 2.49 vs. 2.95 persons, *p* < 0.01), more spent over €400/month on food than in the reference group (30% vs. 24%; median €301–€400 vs. €251–€300). Women with endometriosis were more than twice as likely to report supplement costs exceeding €35/month (22% vs. 9%; median €16–€25 vs. €6–€15; *p* < 0.01). Diet adherence and supplement use were also significantly higher (*p* < 0.01). After controlling for relevant sociodemographic characteristics, endometriosis status remained significantly associated with food expenditure (B = −0.19, β = −0.08, *p* = 0.02) and supplement expenditure (B = −0.52, β = −0.2, *p* < 0.01).

**Conclusions:**

Endometriosis is associated with greater financial burden from dietary self-management. These findings highlight the need for evidence-based nutritional guidance and consideration of economic impact in care.

## 1 Introduction

Endometriosis is a chronic condition characterized by the presence of endometrium-like tissue outside the uterine cavity. Symptoms include pelvic pain, dysmenorrhea, and infertility, among others (De Graaff et al., 2013; Zondervan et al., 2020; (Saunders and Horne, 2021). The precise prevalence of endometriosis is uncertain; however, it is estimated to be between 2% and 10% within the general female population and up to 50% among women struggling with infertility (Becker et al., 2022).

Endometriosis places a substantial healthcare and economic burden on individuals and society. Numerous studies have investigated the costs of this condition; the most comprehensive was the EndoCost study, involving 12 referral centers from 10 countries. This study revealed that the societal and economic burden of endometriosis is comparable to that of diabetes (Simoens et al., 2012). These cost-of-illness studies have focused on the direct healthcare costs of endometriosis, such as medical consultations, surgical interventions, and medications, as well as direct non-healthcare costs and indirect costs associated with work productivity loss (Simoens et al., 2007, Simoens et al., 2011; Soliman et al., 2016; Armour et al., 2019a; Ellis et al., 2022).

At present, there is no definitive treatment for endometriosis. Neither surgical procedures nor medication can be considered a comprehensive solution (Fasolino et al., 2024). Consequently, most affected women use self-management strategies to relieve their symptoms, improve their quality of life, manage their fertility issues, and prevent the condition from recurrence (Armour et al., 2019b; (Spinoni et al., 2025). Nutritional interventions, such as special diets and dietary supplementation, are the most common methods, particularly those with anti-inflammatory and antioxidant properties (Missmer et al., 2010; Karlsson et al., 2020; Van Haaps et al., 2023; Naeini et al., 2025; Dougan et al., 2024; Hearn-Yeates et al., 2025).

Women with endometriosis face a significant financial burden, as non-medical costs, including nutrition-related expenses, are not covered by social insurance systems in most countries. To the best of our knowledge, there has been no specific research on the financial impact of dietary interventions and nutritional supplementation on individuals with endometriosis compared to those without. Our study aimed to fill this gap by using a large sample size to compare the financial burden associated with special diets and dietary supplement use between individuals with endometriosis and those without the condition.

## 2 Methods

### 2.1 Study design and population

A cross-sectional international, parallel-group study was performed within the framework of the Finding Endometriosis using Machine Learning (FEMaLe) Horizon 2020 project to enable the measurement and comparison of the financial implications of nutritional supplementation and special diets incurred by women with medically confirmed endometriosis and a reference group without diagnosed endometriosis. We collected self-reported data through a questionnaire in the freely accessible Lucy mobile health application, which offers robust data security and anonymity features through encryption, safeguarding user information, and maintains confidentiality by not storing personal identifier information or requesting personal data (Balogh et al., 2024). Participants completed the questionnaire in their preferred environment, ensuring real-world relevance and more authentic and representative responses.

Data collection was conducted between 27 December 2023 and 31 August 2024 in Hungary.

A total of 1953 participants of reproductive age were recruited for the study via the Lucy app, which employs the following exclusion criteria: ongoing pregnancy, lactation, and concern for pre-malignancy or presence/history of malignancy.

Of the 1953 eligible participants, 136 were excluded for not completing the questionnaire. Participants who did not provide informed consent for the use of their data in scientific research (*n* = 56) were also excluded, resulting in a sample of 1761. Because the study focused on the Hungarian population, only participants who completed the Hungarian-language version of the questionnaire were included in the final analyses, to minimize potential differences related to country-specific socioeconomic and healthcare factors, resulting in a final sample of 1645. This group was then divided into two cohorts based on their endometriosis status: 524 participants had confirmed a specific date of medical diagnosis for endometriosis, whereas 1121 participants without a medical diagnosis of endometriosis served as the reference group (Fig. 1).

**Fig. 1:**
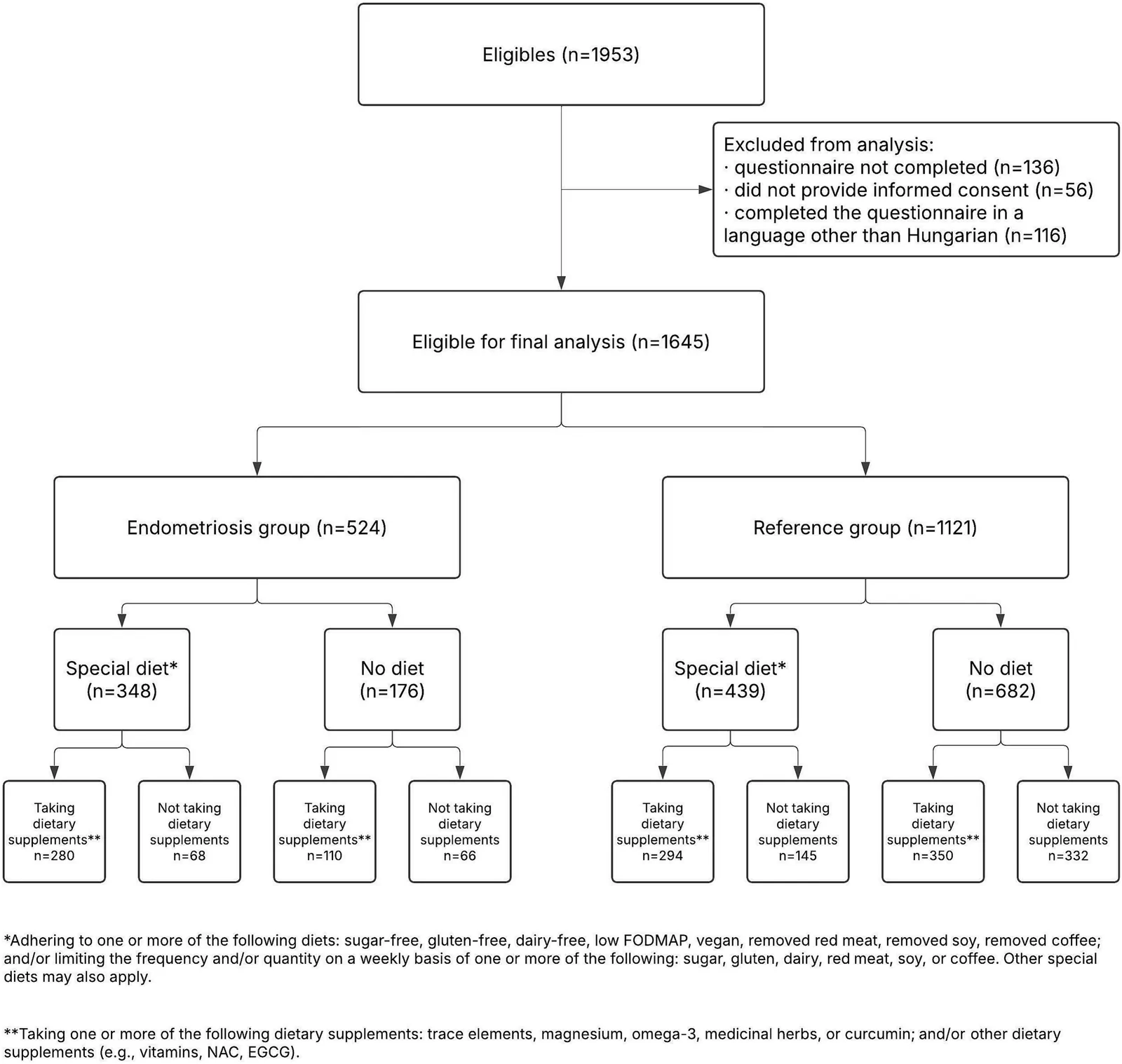
**Study population flow diagram:** Classification by endometriosis status, diet type, and dietary supplement use among individuals aged 14–45 years in Hungary, December 2023–August 2024.

### 2.2 Measures

We developed a self-reported questionnaire to collect data on costs associated with various diets and dietary supplements used by participants.

The Lucy app employs a baseline questionnaire, completed by participants upon their first use of the application.

The study questionnaire consisted of 17 questions and included inquiries regarding demographic data (e.g., the highest level of education attained, number of people living in the respondent's household), diet-related information (including adherence to special diets and their specific type), dietary supplementation, and monthly expenditure on food and nutritional supplements. The questionnaire included single or multiple-choice questions and free-text responses.

The primary outcome was the assessment of potential differences in the financial implications of various dietary supplements and diets between individuals with endometriosis and those without endometriosis.

Secondary outcomes included identifying the most frequently consumed dietary supplements and popular diets in both groups.

Data from Hungarian-speaking respondents, originally provided in Hungarian Forint (HUF), were converted to euros (EUR) at an average exchange rate of 390.66 HUF/EUR, as reported by the European Central Bank for the duration of the questionnaire administration to reflect the economic conditions at the time of data collection and to minimize distortions.

We established predetermined ranges for nutrition-related expenditures and household income levels, and the questions were single-choice items. This methodological decision in survey design served multiple purposes. First, it simplified the response process for participants, as they were not required to recall exact financial figures, which could potentially result in higher response rates. Additionally, when reporting sensitive information such as personal income, even if anonymously, respondents might experience greater ease, as they were not required to disclose exact income figures.

In the case of income, respondents were provided with the ‘prefer not to answer’ option to enhance their comfort level.

### 2.3 Statistical analysis

Continuous variables are summarized as means ± SD and categorical variables as frequencies (%). Normality was tested using the Shapiro-Wilk test. Group comparisons used *t*-tests for normally distributed continuous variables, ANOVA for multiple groups, and Chi-square tests for categorical variables. Two multivariable linear regression models, controlling for age, education, income, and household size, examined associations between endometriosis status and monthly household food and individual supplement expenditures. Outputs include unstandardized (B) and standardized (Beta) coefficients, R^2^, and *p*-values (significance set at *p* < 0.05). Statistical analyses were performed using SPSS Statistics version 17.0 (SPSS Inc., Chicago, IL, USA).

### 2.4 Ethical approval

The study was conducted in accordance with the Declaration of Helsinki, and ethical approval was granted by the Medical Research Council of Hungary (OGYÉI/31355/2021). The study protocol is registered at ClinicalTrials.gov (Identifier: NCT06147687).

## 3 Results

### 3.1 Sociodemographic characteristics of study participants

A total of 1645 women aged 14–45 participated in our study, 524 with a medical diagnosis of endometriosis and 1121 without. The mean age of respondents with reported endometriosis was 33.2 years (SD: 7; *p* < 0.01), whereas the mean age of participants without the condition was 27.8 years (SD: 8; *p* < 0.01).

As for the highest level of education attained, 61% of participants with endometriosis had completed higher or postgraduate education. In comparison, 44% of those without endometriosis achieved this level of education. Regarding the lowest level of education (primary school), the figures were 2% among those with endometriosis and 10% in the reference group (*p* < 0.01).

Women with endometriosis reported living in smaller households (mean: 2.49; SD: 0.98) than those in the reference group (mean: 2.95; SD: 1.33) (*p* < 0.01).

The annual net income of households also differed significantly (*p* < 0.01), with a larger proportion of individuals with endometriosis reporting higher income brackets (Table 1).

**Table 1:** Sociodemographic characteristics: Baseline characteristics of individuals aged 14–45 years with and without endometriosis in Hungary, December 2023–August 2024.

Demographics	Individuals with Medical Diagnosis of Endometriosis (*n* = 524)		Individuals without Medical Diagnosis of Endometriosis (*n* = 1121)		*p*-value
**Mean Age (SD)**	**33.2 (7)**		**27.8 (7.9)**		**<0.01**
**Highest Level of Education**					**<0.01**
Primary School	2%		10%		
Apprenticeship	7%		8%		
Secondary School⁎	5%		8%		
Further Education/College⁎⁎	25%		30%		
Higher Education (University)	56%		40%		
Postgraduate	5%		4%		
**Annual Net Income of the Household**					**<0.01**
Less than €25,000	7%		12%		
€25,001–€35,000	60%		62%		
€35,001–€50,000	25%		21%		
€50,001–€70,000	6%		4%		
More than €70,000	2%		1%		
**Mean Number of People Living in the Household**	**2.49 (0.98)**		**2.95 (1.33)**		**<0.01**
	**Special Diet**⁎⁎⁎	**No Diet**	**Special Diet**⁎⁎⁎	**No Diet**	
	**2.43**	**2.61**	**2.75**	**3.09**	**<0.01**

⁎ (school leaving certificate / GCSE / O levels).

⁎⁎ (A levels / vocational qualifications).

⁎⁎⁎ Adhering to one or more of the following diets: sugar-free, gluten-free, dairy-free, low FODMAP, vegan, removed red meat, removed soy, removed coffee; and/or limiting the frequency and/or quantity on a weekly basis of one or more of the following: sugar, gluten, dairy, red meat, soy, or coffee. Other special diets may also apply.

In a multivariable linear regression evaluating demographic predictors associated with endometriosis status (R^2^ = 0.12, *p* < 0.01; coded as 0 = endometriosis, 1 = reference group), older age (B = -0.02, β = −0.24, *p* < 0.01) and adherence to special diets (B = 0.21, β = 0.22, *p* < 0.01) were independently associated with having endometriosis.

### 3.2 Impact of endometriosis on nutrition-related spending

Our data showed statistically significant differences between the dietary costs of individuals with and without endometriosis (Table 4; Fig. 2). Women with endometriosis on a special diet tended to report higher monthly household food costs despite their household size being smaller than any other group's (mean: 2.43; SD: 0.97, *p* < 0.01), spending more on food than any other group, including those without endometriosis but adhering to a special diet. Among women with endometriosis who followed a special diet, nearly one-third (30%) spent more than €400 per month on food, compared to 24% of the reference group on special diets and only 18% of the reference group not following any diet (*p* < 0.01). The median monthly household food expenditure for the endometriosis group (€301–€400) was significantly higher than the reference group (€251–€300, *p* < 0.01).

**Fig. 2:**
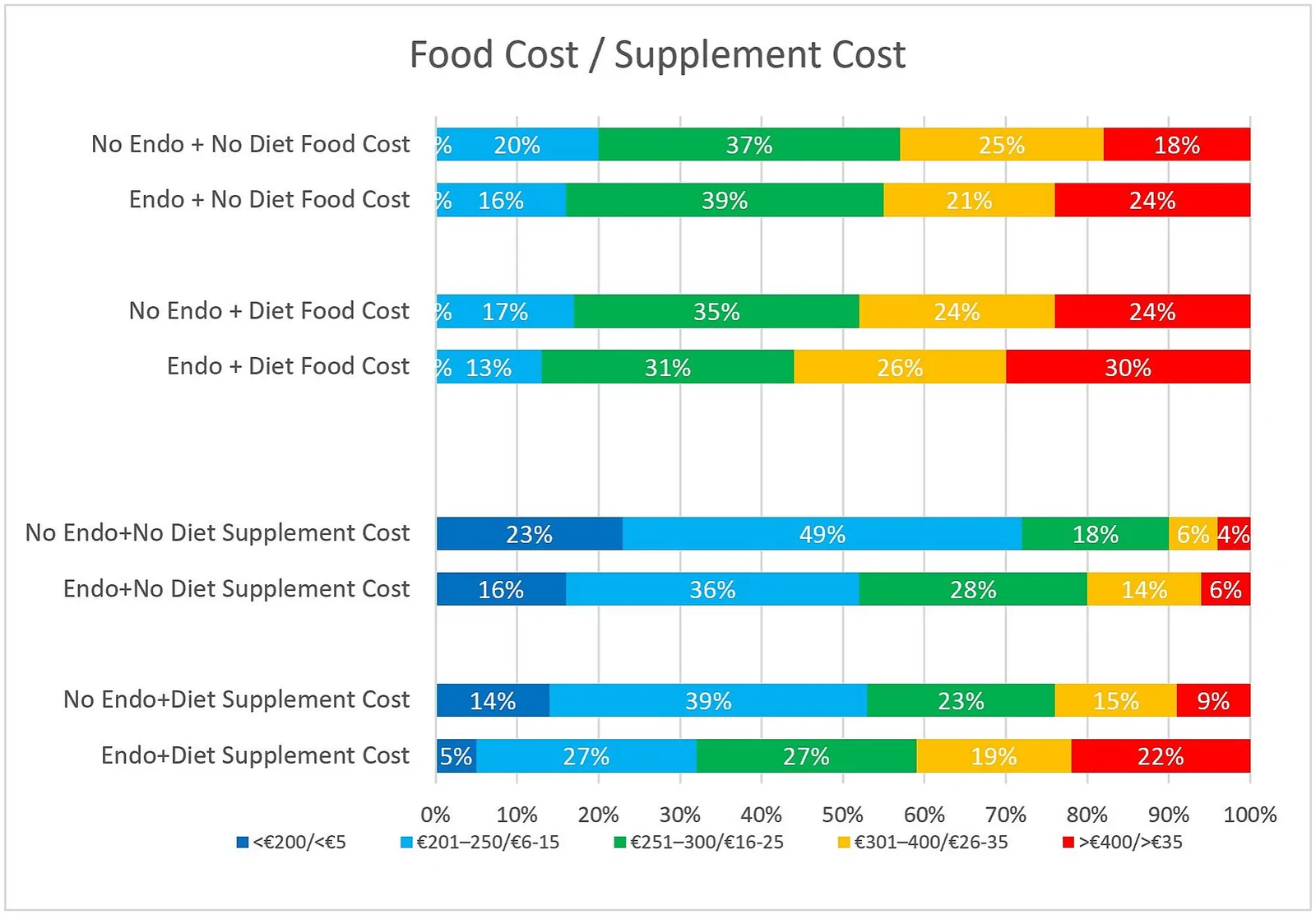
**Distribution of monthly household food and dietary supplement costs:** Comparison by endometriosis status and diet type among individuals aged 14–45 years in Hungary, December 2023–August 2024.

The disparity was even more pronounced in supplement spending. Women with endometriosis on special diets were over twice as likely to report top-tier supplement expenses (>€35/month) as the dieting reference group (22% vs. 9%, *p* < 0.01) (Table 4; Fig. 2). Furthermore, the endometriosis supplement median (€16–€25) was significantly higher than the reference median (€6–€15, *p* < 0.01). These findings align with the group's significantly higher rates of adherence to special diets and supplement use (*p* < 0.01).

Income-stratified analyses revealed statistically significant differences in the middle-income groups. For household food expenditure, the most significant difference occurred in the €35,001–€50,000 income group (*p* < 0.01), where 43% of women with endometriosis spent more than €400 per month compared with 26% of the reference group. Similarly, supplement expenditure was significantly higher in the €25,001–€35,000 and €35,001–€50,000 income groups, with the largest difference in the latter (*p* < 0.01), where 34% of women with endometriosis spent more than €35 per month on supplements compared with 13% of the reference group. No statistically significant differences were observed in the lowest or highest income groups, although women with endometriosis generally reported higher food and supplement expenditure despite living in smaller households (Supplementary Fig. 1, Supplementary Fig. 2, Supplementary Fig. 3, Supplementary Fig. 4, Supplementary Fig. 5).

To test whether expenditure differences persisted after accounting for sociodemographic disparities, we ran adjusted multivariable linear regression models for both outcome variables. In the adjusted model for monthly household food expenditure (R^2^ = 0.18, *p* < 0.01), household size (B = 0.33, β = 0.38 *p* < 0.01), household net income (B = 0.30, β =0.22, *p* < 0.01), age (B = 0.01, β = 0.099, *p* < 0.01), and level of education (B = 0.07, β = 0.08, *p* < 0.01) were all significantly associated with higher spending. Nevertheless, endometriosis status remained a statistically significant independent predictor of higher food expenditure (B = -0.19, β = −0.08, *p* = 0.02). For individual supplement expenditure (R^2^ = 0.07, *p* < 0.01), age (B = 0.01, β = 0.09, *p* = 0.03) and net income (B = 0.13, β = 0.08, *p* = 0.03) were positively linked to costs, but endometriosis status proved to be the primary overall predictor (B = -0.52, β = −0.20, *p* < 0.01).

## 4 Discussion

Our study found significant differences in special diet and dietary supplement expenditure between participants with and without endometriosis. Our findings suggest that women with endometriosis, particularly those following a special diet, tend to have higher food and supplement costs than those without the condition (Table 4; Fig. 2). Women with endometriosis on a special diet reported the highest monthly food costs among the examined groups, despite their average household size being the smallest. Similarly, they spent more on dietary supplements than any other cohort examined, suggesting that nutritional interventions for endometriosis may contribute to a disproportionate financial burden (Table 4; Fig. 2). These differences were significant in the middle-income groups, indicating that the additional financial burden associated with nutritional self-management is not confined to the highest-income households. In addition, women with endometriosis tended to be older, more highly educated, and live in smaller households with higher income levels than those without the condition (Table 1).

Importantly, adjusting for sociodemographic variables suggests that these financial disparities may not be explained solely by baseline demographic differences. While household structure and socioeconomic standing would be expected to influence routine living expenses, endometriosis status remained an independent driver of food costs and a primary determinant of supplement spending − outweighing the effects of both age and income. Together with the finding that older age and dietary self-management characterize this population, these results may indicate that the higher financial burden is more closely related to disease-specific self-management than to underlying sociodemographic factors.

Existing research has shown that endometriosis imposes a substantial economic burden globally (Darbà and Marsà, 2022). However, most studies have concentrated on direct healthcare costs (e.g., surgery, hospitalization, outpatient care, medication) and indirect costs associated with productivity loss resulting from absenteeism and presenteeism, while other components of the financial burden of endometriosis − especially out-of-pocket expenses of patient self-management − remain relatively underexplored.

Indirect costs have been shown to exceed direct healthcare costs in most settings (Soliman et al., 2016). A U.S. study reported annual direct costs of USD 16573 per patient with incremental direct costs of USD 10002 and additional indirect costs of about USD 2000 due to reduced work productivity (Soliman et al., 2018) Similarly, in another study, annual direct costs of USD 12118 and indirect costs of about USD 16000 per patient were estimated (Ellis et al., 2022) In an Australian study, the annual cost per woman ranged between Int $16,970 and $20,898, with 75–84% due to productivity losses (Armour et al., 2019a).

European studies have reported similar findings. Annual costs of €9579 per woman have been documented, with two-thirds of these costs attributed to productivity loss and one-third to healthcare utilization (Simoens et al., 2007, 2012). In Hungary, a recent study has shown an increase in inpatient care costs and national health insurance expenditures for endometriosis between 2010 and 2019, particularly among younger women (Pónusz-Kovács et al., 2024).

To the best of our knowledge, there are very few data on the economic burden of the nutritional aspects of endometriosis. One study, comprising 250 participants and lacking a reference group, was conducted solely in Germany, with no specified diets (Heinze et al., 2024). According to their findings, 62.8% (*n* = 157) of participants spent €293.45 (SD: €321.43) per person yearly on food supplements. Diet adaptation costs were incurred by 50.8% (*n* = 127), averaging €376.18 (SD: €333.07) over 12 months. For the entire study population, average nutrition-related spending was €387.19 (SD: €492.75) per capita per year.

Women with endometriosis often seek alternative solutions to manage their symptoms or prevent the recurrence of the disease, an important element of which is nutrition. This involves both special diets and nutritional supplements. These women are likely to be more health-conscious, and the psychological factor of managing a chronic condition through a strict diet and thus feeling empowered by taking an active role in their own treatment may also contribute to their increased willingness to spend more on dietary modifications and supplements, despite inconsistent evidence supporting the efficacy of nutritional interventions in endometriosis.

We found that a substantial proportion of women with endometriosis adhered to one or more special diets, with only 34% of the respondents reporting that they did not follow any; this figure was 60% in the reference group (Table 2). Assessing research on the most frequently reported restricted nutrients in our survey suggests that evidence for their efficacy remains inconsistent.

**Table 2:** Comparison of dietary modifications: Prevalence and types of dietary modifications among individuals aged 14–45 years with and without endometriosis in Hungary, December 2023–August 2024.

Type of Diet	Individuals with Medical Diagnosis of Endometriosis	Individuals without Medical Diagnosis of Endometriosis	p-value
	(n = 524)	(n = 1121)	
**Sugar**			**<0.01**
Limited	24%	21%	
Removed	46%	20%	
**On Diet Total**	**70%**	**41%**	
**Red Meat**			**<0.01**
Limited	23%	13%	
Removed	38%	26%	
**On Diet Total**	**61%**	**39%**	
**Dairy**			**<0.01**
Limited	26%	18%	
Removed	32%	21%	
**On Diet Total**	**58%**	**39%**	
**Soy**			**<0.01**
Limited	16%	8%	
Removed	38%	31%	
**On Diet Total**	**54%**	**39%**	
**Gluten**			**<0.01**
Limited	11%	7%	
Removed	33%	19%	
**On Diet Total**	**44%**	**26%**	
**Coffee**			**<0.05**
Limited	19%	13%	
Removed	17%	15%	
**On Diet Total**	**36%**	**28%**	
**Other Diets**			
**Vegan (On Diet Total)**	7%	5%	**<0.05**
**Low FODMAP (On Diet Total)**	5%	1%	**<0.01**
**Other Diet (On Diet Total)**	17%	10%	**<0.01**
**Not on Diet**	**34%**	**60%**	**<0.01**

In our survey, the most prevalent special diet was one removing or limiting sugar (70% vs 41%). Most studies have not analyzed the effects of sugar as an individual nutrient on the pain and quality of life of women with endometriosis ((Schwartz et al., 2022); Huijs and Nap, 2020; Van Haaps et al., 2023). One study associated a higher glycemic index with an increased risk of laparoscopically confirmed endometriosis (Schwartz et al., 2022).

Evidence regarding red meat restriction (61% vs 38% in our study) is contradictory, with some studies finding an association between higher consumption of red meat and increased endometriosis risk (Parazzini et al., 2004; Yamamoto et al., 2018); however, the effect is not consistently evident (Parazzini et al., 2004, (Parazzini et al., 2013); Yamamoto et al., 2018; Simmen and Kelley, 2018).

Dairy products were limited or excluded by 58% of women with endometriosis on a special diet, as opposed to 38% of the reference group. Dairy is a similarly controversial nutrient in endometriosis (Krabbenborg et al., 2021; Van Haaps et al., 2023); some studies indicated neutral (Parazzini et al., 2004) or even protective effects (Nodler et al., 2019), while others suggested perceived symptom improvement after the restriction of dairy products (Krabbenborg et al., 2021).

Other dietary modifications – such as low-FODMAP, gluten-free, vegan diets, and diets excluding coffee or soy – were frequently reported. Multiple studies have explored the effects of various nutrients on endometriosis, considering both the preventive and therapeutic aspects, with contradictory results (Krabbenborg et al., 2021; Mitsunami et al., 2024).

Besides special diets, nutritional supplements are widely used in the self-management of endometriosis. One study found that women with endometriosis were more likely to use vitamins and minerals compared to those without endometriosis (OR = 1.72; 95% CI: 1.24, 2.38), which aligns with our findings (Fisher et al., 2016) (Table 3). Specifically, omega-3 use was 16 percentage points higher among women with endometriosis than in the reference group. This difference was 15 percentage points for trace elements and vitamins, 14 percentage points for magnesium and 10 percentage points for medicinal herbs. Notably, curcumin use among women with endometriosis was five times more common than among those without endometriosis (15% vs 3%).

**Table 3:** Comparison of dietary supplement use: Prevalence and patterns of dietary supplement use among individuals aged 14–45 years with and without endometriosis in Hungary, December 2023–August 2024.

Type of Dietary Supplement	Individuals with Medical Diagnosis of Endometriosis (n = 524)		Individuals without Medical Diagnosis of Endometriosis (n = 1121)		p-value
Magnesium	56%		42%		<0.01
Trace Elements	36%		21%		<0.01
Omega-3	31%		15%		<0.01
Medicinal Herbs	29%		19%		<0.01
Curcumin	15%		3%		<0.01
Other (e.g., vitamins)	88%		74%		<0.01
Not Taking Any Supplements	25%		44%		<0.01
**Mean Number of Dietary Supplements Taken (SD)**	1.66 (1.37)		0.99 (1.14)		**<0.01**
	Special Diet*	**No Diet**	Special Diet*	**No Diet**	
	1.89 (1.4)	1.20 (1.2)	1.32 (1.25)	0.79 (1.01)	**<0.01**
**Proportion of Individuals Taking Dietary Supplements by Diet and Endometriosis Status**	80%	62%	67%	49%	**<0.01**

*Adhering to one or more of the following diets: sugar-free, gluten-free, dairy-free, low FODMAP, vegan, removed red meat, removed soy, removed coffee; and/or limiting the frequency and/or quantity on a weekly basis of one or more of the following: sugar, gluten, dairy, red meat, soy, or coffee. Other special diets may also apply.

**Table 4:** Monthly household food and dietary supplement costs: Cost estimates stratified by endometriosis status and diet type among individuals aged 14–45 years in Hungary, December 2023–August 2024.

Dietary Costs and Dietary Supplementation Costs	Individuals with Medical Diagnosis of Endometriosis (n = 524)		Individuals without Medical Diagnosis of Endometriosis (n = 1121)		p-value
	Special Diet (*n* = 348)	No Diet (*n* = 176)	Special Diet (*n* = 439)	No Diet (*n* = 682)	
**Household Food Cost/Month**					**<0.01**
Below €200	0%	0%	0%	0%	
€201–€250	13%	16%	17%	20%	
€251–€300	31%	39%	35%	37%	
€301–€400	26%	21%	24%	25%	
More than €400	30%	24%	24%	18%	
**Dietary Supplement Cost/Month**					**<0.01**
Less than €5	5%	16%	14%	23%	
€6–€15	27%	36%	39%	49%	
€16–€25	27%	28%	23%	18%	
€26–€35	19%	14%	15%	6%	
More than €35	22%	6%	9%	4%	

Overall, evidence concerning the efficacy of nutritional supplements remains contradictory, with many studies limited by small sample sizes, short trial durations, and the predominance of animal or in vitro trials (Krabbenborg et al., 2021; (Parazzini et al., 2013); Nodler et al., 2020). Further research is required to address these limitations. Moreover, accounting for placebo and nocebo effects in human studies would be crucial to precisely assess the efficacy of nutritional interventions in endometriosis.

Strengths of our research include a large sample size and a reference group for comparative analysis. We collected self-reported, real-world data through a mobile app, which helped reduce selection bias. At the same time, app-based recruitment may still introduce bias toward digitally literate, health-conscious users with smartphone access.

Limitations to our study include that we had no access to medical records; the date of medical diagnosis was self-reported, which may introduce reporting bias. The use of predetermined ranges for food and supplement expenditure and income data, while potentially increasing response rate, may limit the precision of financial analysis. As our study was cross-sectional, the lack of longitudinal data did not allow for the assessment of dietary habits or costs over time.

## 5 Conclusion

Our study demonstrates that dietary self-management among women with endometriosis is associated with higher monthly household food expenditures and individual dietary supplement spending, often despite inconsistent evidence of their efficacy.

Crucially, multivariable linear regression models suggest that these higher food and supplement expenditures remain independently associated with endometriosis status even after adjusting for key sociodemographic confounders.

These increased expenditures were significantly higher in middle-income groups, while differences were not statistically significant in the lowest and highest income categories. Future research should evaluate the efficacy of special diets and dietary supplements in endometriosis, including synergistic, placebo, and nocebo effects. The costs associated with specific individual nutritional interventions and their potential impact on healthcare costs also need to be assessed. A better understanding of both clinical and economic consequences of nutritional interventions in endometriosis could lead to evidence-based recommendations and policy development.

The following are the supplementary data related to this article.

**Supplementary Fig. 1:**
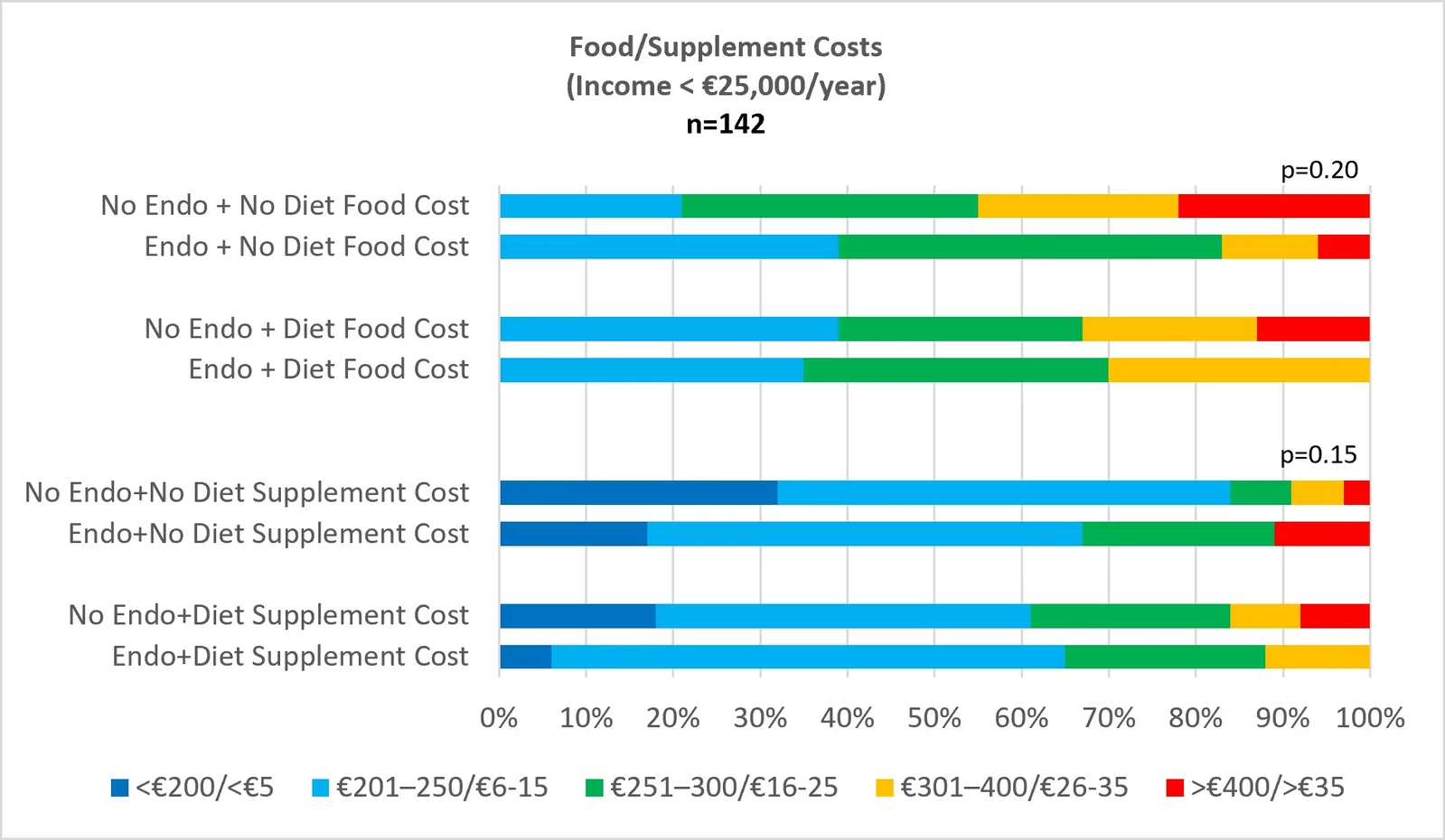

**Supplementary Fig. 2:**
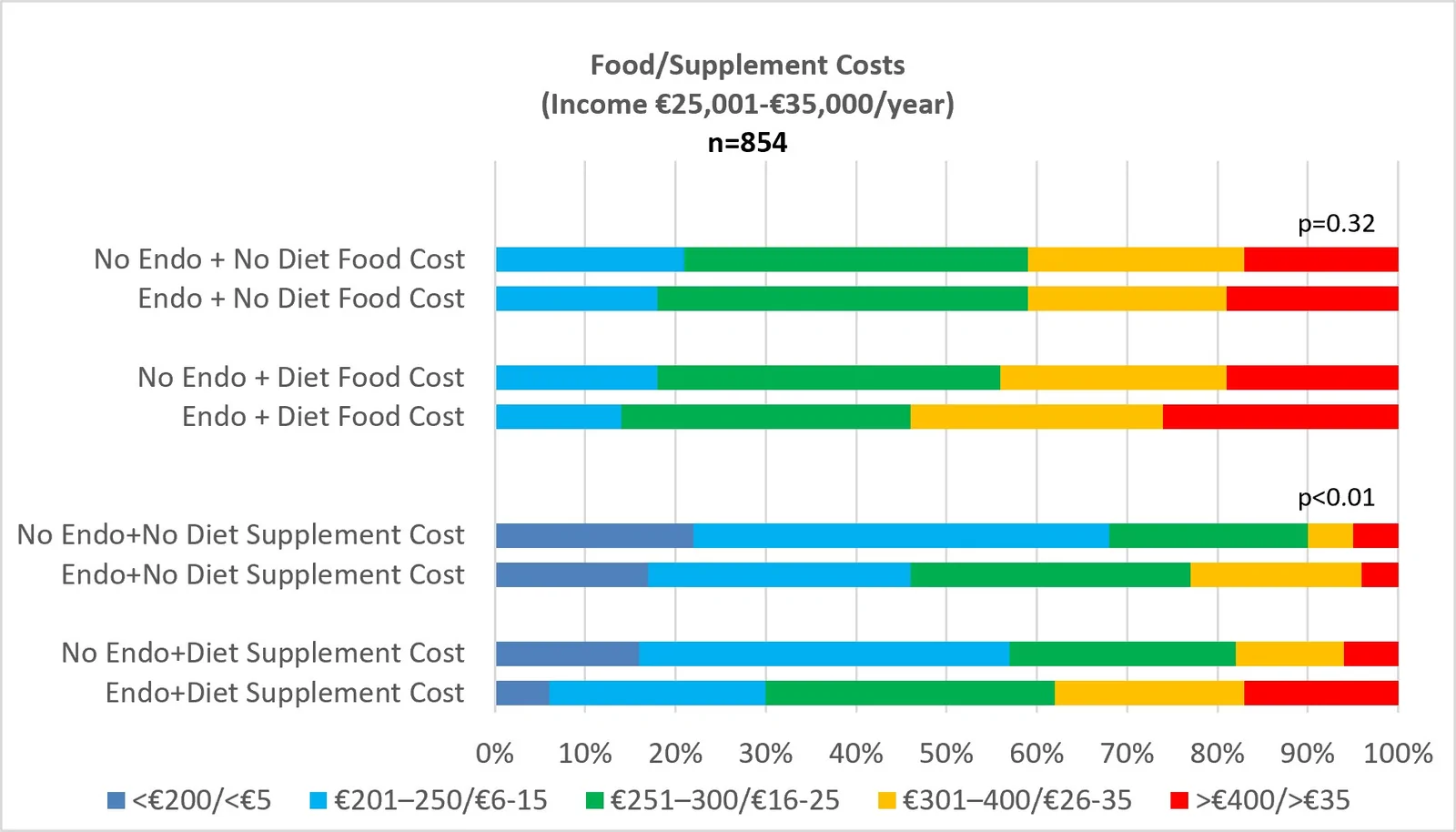

**Supplementary Fig. 3:**
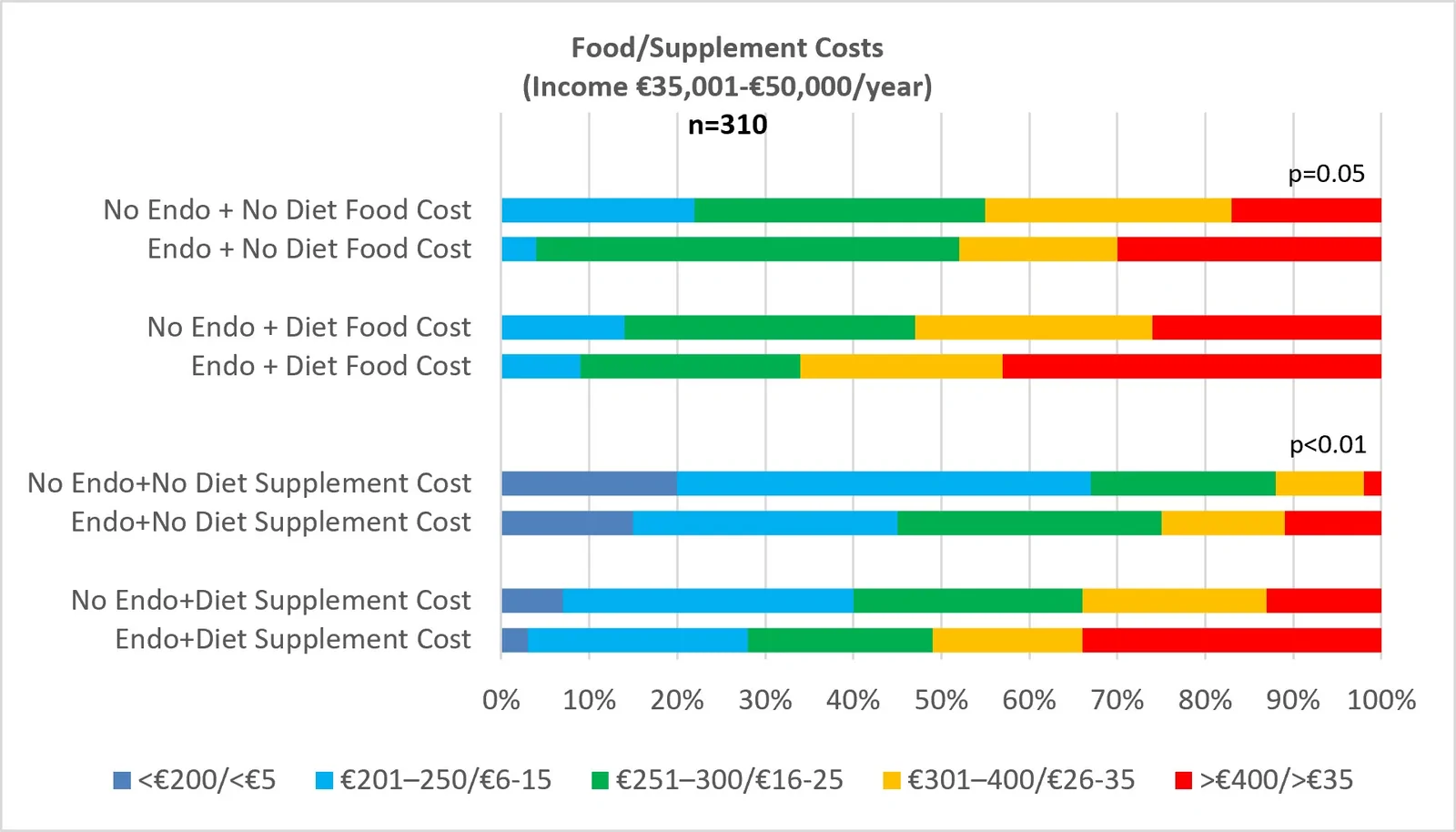

**Supplementary Fig. 4:**
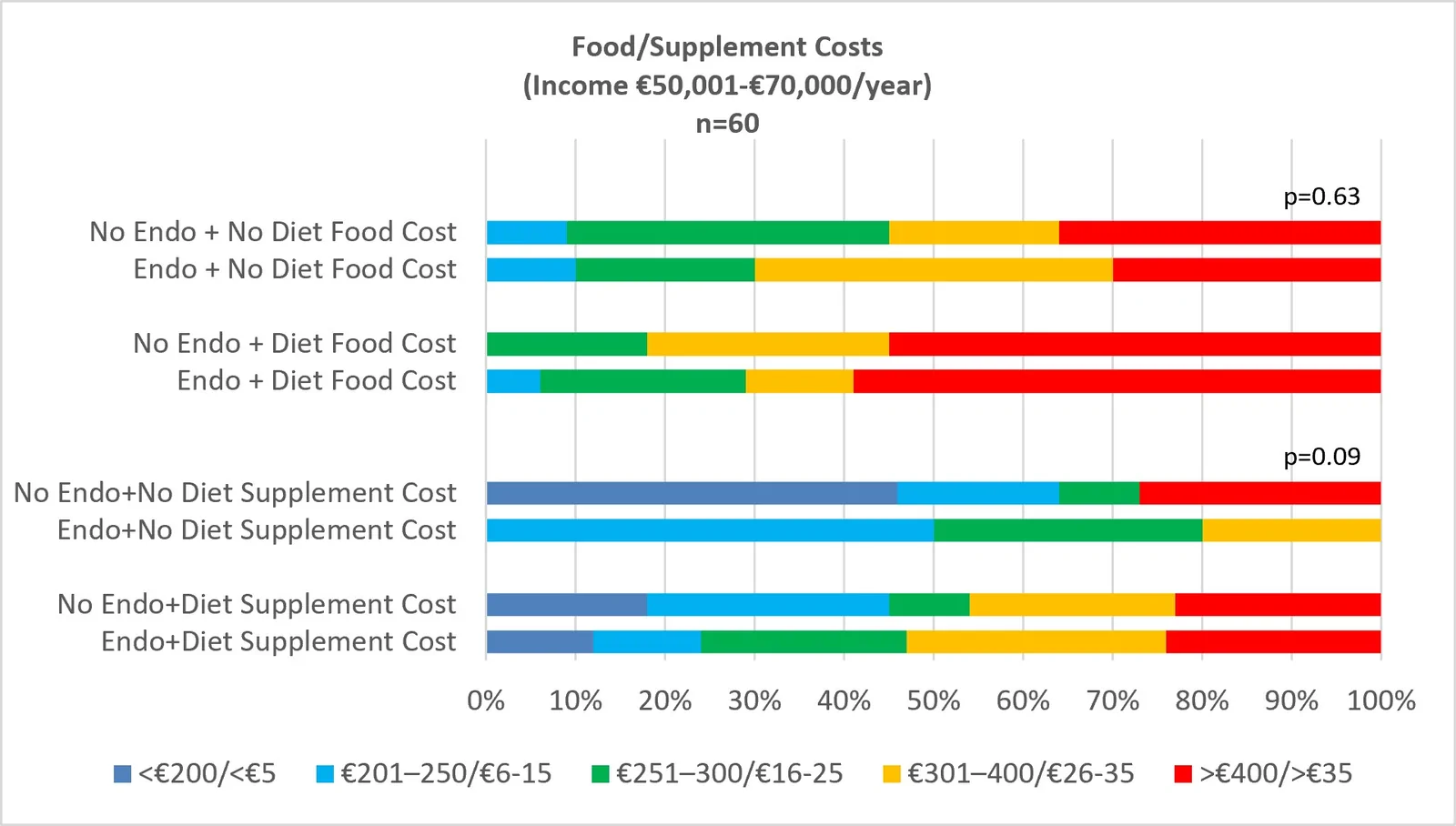

**Supplementary Fig. 5:**
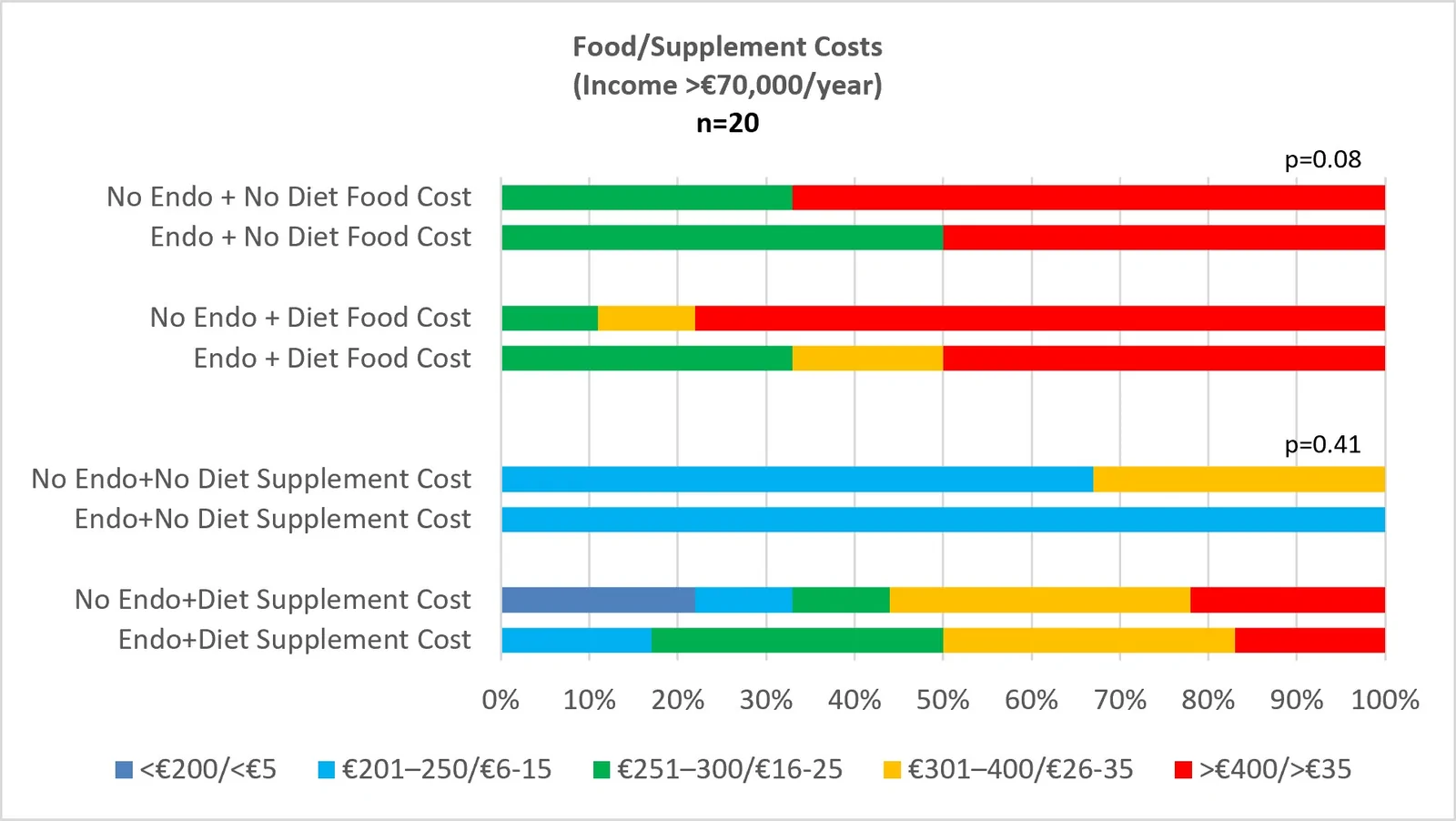

## Declaration of generative AI and AI-assisted technologies in the manuscript preparation process

During the preparation of this work the authors used ChatGPT (OpenAI) for language editing and grammar refinement. After using this tool, the authors reviewed and edited the content as needed and take full responsibility for the content of the published article.

## CRediT authorship contribution statement

**Linda Balpataki:** Writing – review & editing, Writing – original draft, Resources, Conceptualization. **Dora B.Balogh:** Writing – review & editing, Resources. **Gernot Hudelist:** Writing – review & editing. **Christian M.Becker:** Writing – review & editing. **Andrew W.Horne:** Writing – review & editing. **Dominika Miklos:** Writing – review & editing. **Nandor Acs:** Writing – review & editing. **Attila Bokor:** Writing – review & editing, Writing – original draft, Resources, Funding acquisition, Conceptualization.

## Funding sources

This study is part of the project Finding Endometriosis using Machine Learning (FEMaLe), which has received funding from The European Union's Horizon 2020 research and innovation program (Grant No. 101017562). The funders had no role in study design, data collection and analysis, decision to publish, or preparation of the manuscript. This research was also supported by the János Bolyai Research Scholarship of the 10.13039/501100003825Hungarian Academy of Sciences (BO/00147/25/5).

## Declaration of competing interest

The authors declare the following financial interests/personal relationships which may be considered as potential competing interests: Andrew W. Horne’s institution received grant funding from EU, UKRI, NIHR, CSO, Wellbeing of Women and Roche Diagnostics and consultancy fees from Roche Diagnostics, Gesynta, and Theramex. Andrew W. Horne received honoraria for lectures from Gedeon Richter and Theramex. Andrew W. Horne is listed as co-inventor on patent application (UK Patent Application No. 2217921.2, International Patent Application No. PCT/GB2023/053076). Andrew W. Horne is President of the World Endometriosis Society, Co-editor in chief of Reproduction and Fertility, Trustee and Medical Advisor to Endometriosis UK, and Specialty Advisor to the Scottish Government’s Chief Medical Officer for Obstetrics and Gynaecology. Nandor Acs received consultancy fees from Gedeon Richter. Attila Bokor’s institution received consultancy fees from Gedeon Richter, Ethicon, and Medtronic.

## Data Availability

Data will be made available on request.
